# Constitutive Activation of Ectodermal β-Catenin Induces Ectopic Outgrowths at Various Positions in Mouse Embryo and Affects Abdominal Ventral Body Wall Closure

**DOI:** 10.1371/journal.pone.0092092

**Published:** 2014-03-19

**Authors:** Xuming Zhu, Sixia Huang, Lingling Zhang, Yumei Wu, Yingwei Chen, Yixin Tao, Yushu Wang, Shigang He, Sanbing Shen, Ji Wu, Baojie Li, Xizhi Guo, Lin He, Gang Ma

**Affiliations:** 1 Bio-X Institutes, Key Laboratory for the Genetics of Developmental and Neuropsychiatric Disorders (Ministry of Education), Shanghai Jiao Tong University, Shanghai, PR China; 2 Department of Dermatology, Luwan Branch, Ruijin Hospital, Shanghai Jiao Tong University School of Medicine, Shanghai, PR China; 3 Department of Biomedical Engineering, Shanghai Jiao Tong University, Shanghai, PR China; 4 Regenerative Medicine Institute, School of Medicine, National University of Ireland Galway, Newcastle Road, Galway, Ireland; National Cancer Center, Japan

## Abstract

Vertebrate limbs originate from the lateral plate mesoderm (LPM) and the overlying ectoderm. While normal limb formation in defined regions has been well studied, the question of whether other positions retain limb-forming potential has not been fully investigated in mice. By ectopically activating β-catenin in the ectoderm with Msx2-cre, we observed that local tissue outgrowths were induced, which either progressed into limb-like structure within the inter-limb flank or formed extra tissues in other parts of the mouse embryo. In the presumptive abdominal region of severely affected embryos, ectopic limb formation was coupled with impaired abdominal ventral body wall (AVBW) closure, which indicates the existence of a potential counterbalance of limb formation and AVBW closure. At the molecular level, constitutive β-catenin activation was sufficient to trigger, but insufficient to maintain the ectopic expression of a putative limb-inducing factor, Fgf8, in the ectoderm. These findings provide new insight into the mechanism of limb formation and AVBW closure, and the crosstalk between the Wnt/β-catenin pathway and Fgf signal.

## Introduction

In vertebrates, the lateral plate mesoderm (LPM) is located in the flank of the embryonic trunk and gives rise to the mesoderm of multiple organs and tissues, which includes limbs and the abdominal ventral body wall (AVBW).

Specification of the prospective limb territories in LPM requires the participation of the homeobox and Tbx genes [1]–[4]. Subsequently, the LPM cells undergo continuous proliferation and bulge outwards, and express Fgf10, inducing the overlying ectoderm to thicken in the most distal region, forming a structure known as the apical ectodermal ridge (AER), in turn producing Fgf8 to maintain the expression of Fgf10 in the underlying LPM [5]. Deficiency of Fgf signal causes severe limb truncation or complete loss of limb formation [6], [7].

In addition to the Fgf gene family, Wnt proteins are involved in the limb initiation. In chicks, Wnt2b and Wnt8c are expressed in the LPM of the presumptive forelimb and hindlimb regions, respectively, where they could both directly induce the expression of Fgf10 [8]. Moreover, another Wnt protein, Wnt3a, acts downstream of Fgf10 to activate and maintain the expression of Fgf8 in the AER [8]. All of these Wnt members exert their function through the β-catenin-dependent canonical Wnt pathway because enforced β-catenin expression is sufficient to induce the expression of Fgf10 in the LPM, and β-catenin deletion abolishes the expression of Fgf8 in the AER [8], [9]. However, in mouse embryos, no individual Wnt member has been observed to be expressed in the LPM before limb initiation to trigger Fgf10 expression, but after limb bud forms, Wnt3 that is expressed in the limb ectoderm, indeed maintains Fgf8 expression in the AER, serves the same function as the chick homolog of Wnt3a [9].

Certain Fgf and Wnt family members are putative limb-inducing factors because of their ability to induce ectopic AER or extra limb formation in both chick and mouse embryos. In chick embryos, Fgfs such as Fgf4, 7, 8 and 10, induce ectopic formation of AER in the flank and dorsal trunk posterior to the 10^th^ somite [10]. Ectopic expression of Wnt2b and Wnt8c also results in extra limb in the inter-limb flank by activating Fgf signal [8]. In mouse embryos, exogenous Fgf4 bead application can induce ectopic AER along the body flank [11], but no Wnt protein has been shown to induce extra limb formation. However, constitutive β-catenin activation in the ectoderm induces ectopic Fgf8-expressing AER in the inter-limb flank and extra limbs in the abdomen [12].

The abdomen of the mouse embryo is first covered by the primary body wall, consisting of an epithelial membrane. Beginning in E12, the secondary body wall, comprising LPM-derived mesoderm and the overlying ectoderm, begins to form and moves from the lateral side to the midline of the abdomen to replace the primary body wall. At E14.5, an obvious umbilical ring structure can be observed in the center of the abdomen surface to facilitate the development of gut. By E16.5, the secondary body wall from each lateral side has fused at the midline, and the AVBW has closed around the umbilical vessels [13].

Although no clear definition of the relationship between limb formation and AVBW closure has been reported, it is not difficult to speculate that the two processes are tightly coordinated, considering that they are both originated from the LPM. Mouse models of various gene knockouts, including TFAP2α chimeric mice and Alk3 conditional knockout mice, show both limb defects and ventral body wall deformities [14], [15]. Moreover, a human congenital disease, Limb-body wall-complex, is also characterized by deformed limbs and an abnormal ventral body wall [16].

For a broader assessment of the limb-forming potential in mouse embryos, we activated ectodermal β-catenin through Msx2-cre, which showed obvious activity in the ectoderm at different various positions, including the flank, head, and tail regions. We observed that constitutive ectodermal β-catenin activation not only induced ectopic limb buds in inter-limb abdomen but also gave rise to extra outgrowths in the head and tail regions, most likely by triggering the expression of Fgf8. In addition, we observed that the ectopic formation of limb in the abdomen was coupled with defective AVBW closure in severely affected Msx2-cre; β-catenin^Δex3^ mutants, whereas in Msx2-cre; β-catenin cKO mutants disrupted limb formation correlated with premature AVBW closure.

## Materials and Methods

### Mice embryo collection and RNA in situ hybridization

Mice of Msx2-cre [17], β-catenin^fl^ [18], β-catenin (ex3)^fl^ [19] were maintained in an SPF environment. All animal experiments in this study were conducted in accordance with the National Research Council Guide for Care and Use of Laboratory Animals, according to the approved protocols of the Institutional Animal Care and Use Committee of Shanghai, China [SYXK (SH) 2011–0112]. Efforts were made to minimize suffering. Embryos at the indicated stages were collected and fixed with 4% paraformaldehyde/PBS overnight at 4 °C. Whole-mount RNA in situ hybridizations were performed as previously described [17], using the probes for Fgf8, Msx1, Shh, Hoxd13, and Hoxa11 [17].

### H&E, Alcian blue and Alizarin red staining

Paraffin-sectioned tissue was rehydrated with water and stained with hematoxylineosin for 5 minutes, and placed under running tap water to remove excess dye. After staining the sample with eosin for 30 seconds, ethanol was used to remove excess eosin solution and dehydrate the sections. Subsequently, the tissue was cleared with xylene and mounted. For bone staining, fresh embryos at E16.5 were skinned, eviscerated, and fixed with 95% ethanol for 2 days and transferred to acetone for 1 day. The embryos were then placed in staining solution, containing 0.015% Alcian blue and 0.005% alizarin red for 1 week, and then treated with 1% KOH until clear.

### β-Galactosidase staining

Embryos were fixed for 10–30 min at room temperature in 4% paraformaldehyde/PBS depending on their developmental stage, then washed once in PBS and placed in X-gal staining solution, containing 1 mg/mL X-gal in N,N-dimethylformamide, 5 mM K_3_Fe(CN)_6_, and 5 mM K_4_Fe(CN)_6_ overnight at room temperature. Stained tissues were washed twice in PBS, post-fixed for 1 hour at room temperature with 4% paraformaldehyde/PBS, and washed again in PBS before documentation.

## Results

### Ectodermal activation of β-catenin caused multiple defects in the mouse embryo

**Figure 1:**
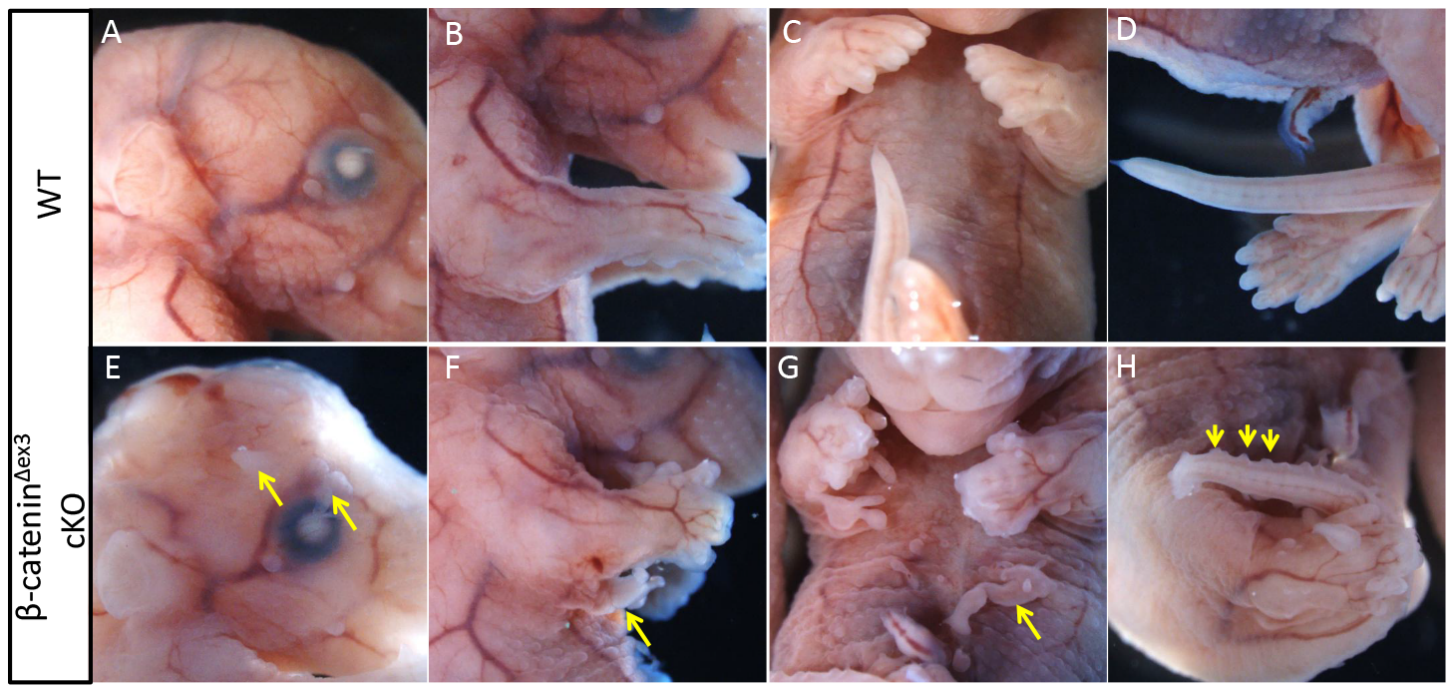
Ectopic outgrowth formation was observed at various positions in Msx2-cre; β-catenin^Δex3^ mutants at E16.5. (A and E) Ectopic tissues were observed above the eyes in the mutants (yellow arrows). (B and F) An extra limb was formed immediately posterior to the forelimb (yellow arrow). (C and G) Ectopic outgrowths appeared in the abdominal region (yellow arrow). (D and H) The mutants showed saw-like tails due to the formation of ectopic tissues on the tail surface (yellow arrowheads).

The activity of Msx2-cre was previously shown present in limb ectoderm and inter-limb flank ectoderm [9], [20]. We first re-examined Msx2-cre activity by whole-mount β-galactosidase staining of Msx2-cre; Rosa26 embryos at E10.5. The activity of Msx2-cre was not confined to limb and inter-limb ectoderm, but was also present in the embryonic head region and tail bud (Fig. S1). Thereafter, we mated Msx2-cre mice with β-catenin^ex3^ floxed mice. The Msx2-cre; β-catenin^Δex3^ mutants died immediately after birth, most likely because of the exencephaly phenotype. The result of skeleton analysis suggested that a severely malformed skull was responsible for the exencephaly (Fig. S2A and D). No other skeleton deformities were observed, with the exception of the limb skeleton, which is characterized by a hypomorphic zeugopod and hyperplastic autopod. The zeugopod skeletal elements of mutant limbs were generally shortened, and were sometimes non-calcified (Fig. S2B, C, E, and F). In mutant autopods, not only pre-axial polydactyly and post-axial polydactyly were observed, but ectopic digits could be found along the dorsal-ventral axis (Fig. S2G–I). In addition, digit ossification was also impaired (Fig. S2B, C, E and F). Apart from these skeletal defects, the most obvious phenotype was extra outgrowths formation, which varied in size and shape, and could be observed in multiple positions along the embryo, which includes the head, ventral trunk, and tail (Fig. 1A–H). In the abdomen, all mutants developed such outgrowths (Fig. 1F and G), and approximately 30% of the mutants possessed extra limbs in the inter-limb region. In mutant tails, extra tissues caused the tail to resemble a saw (Fig. 1H).

### Ectopic outgrowths expressed Fgf8, Msx1 and Shh at multiple positions in the mouse embryos

**Figure 2:**
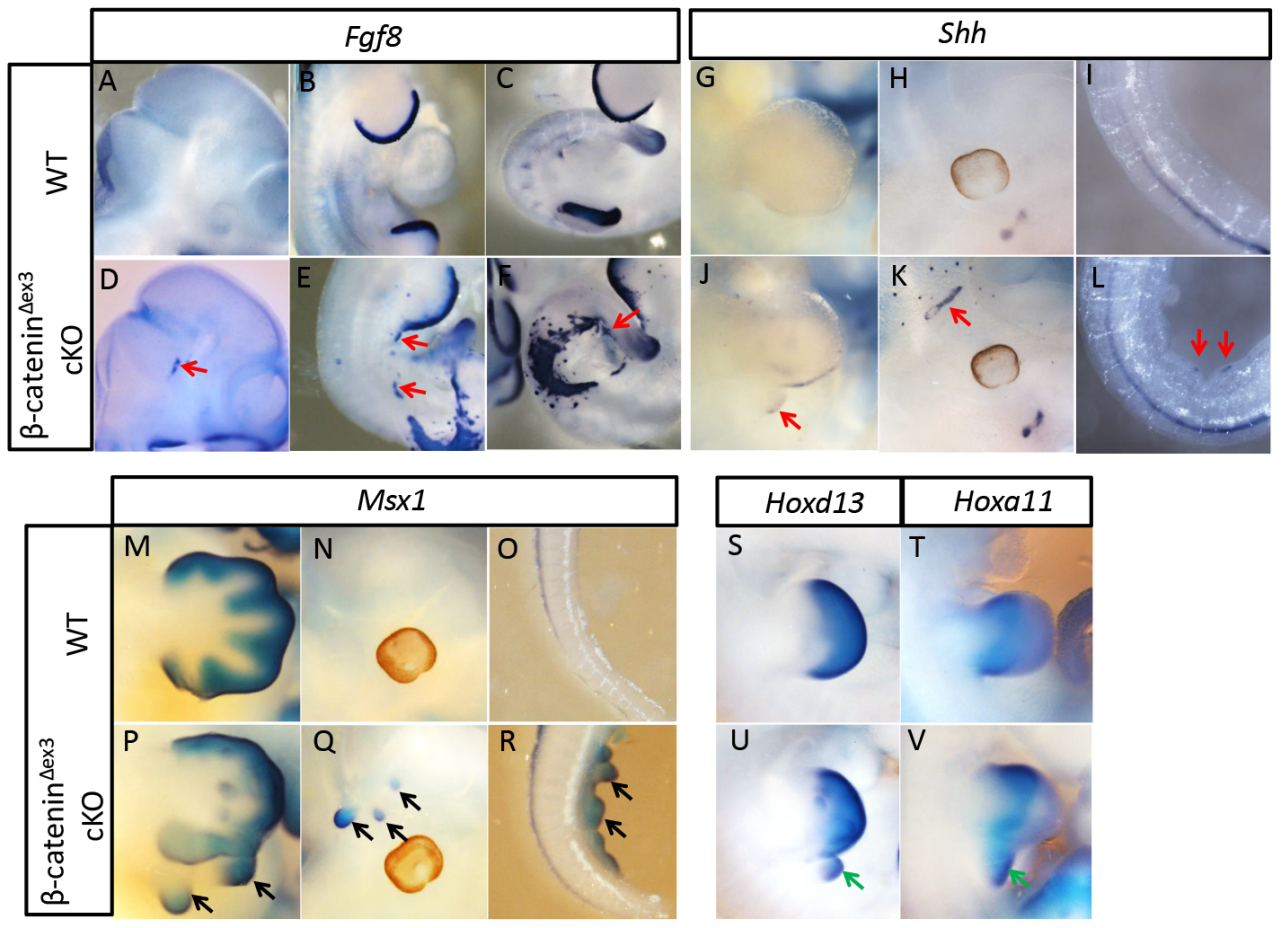
The molecular identity of ectopic outgrowths. (A–F) The expression of ectopic Fgf8 was observed in the head, inter-limb flank, and tail regions at E10.5 (red arrowheads). (G–L) The expression of Shh was also detected in these extra tissues at E11.5 (red arrowheads). (M–R) The expression of Msx1 was observed in the extra tissues at the lateral flank, head, and tail regions at E12.5 (black arrowheads). (S–V) Only extra limbs immediately adjacent to the forelimb showed normal pattern identity, as reflected by the expression of Hoxd13 and Hoxa11 (green arrowheads), marking the autopod and zeugopod regions of the limb bud, respectively.

We then investigated whether ectopic outgrowths observed in other positions, such as the head and tail, also possessed limb bud identity as well. We observed that Fgf8 was expressed in all outgrowth-forming positions at E10.5, which corresponded to cre activity (Fig. 2A–F). Shh expression, indicating the polarizing activity in limb buds, was also observed in these ectopic tissues (Fig. 2G–L). Msx1, whose expression could be found in the undifferentiated mesenchyme in distal limbs, was also detected in all these ectodermal outgrowths (Fig. 2M–R). These results suggested that the ectopic outgrowths had certain characteristics of early stage limb buds. However, only extra limb buds immediately adjacent to normal limb buds in the LPM region expressed Hoxd13 and Hoxa11, markers for the autopod and zeugopod, respectively (Fig. 2S–V). No expression of such genes was observed in other extra outgrowths (data not shown), which indicated that the development of precisely patterned limbs requires additional factors, which are provided by the presumptive limb-forming regions.

### Expression of Fgf8 was triggered but not maintained in the ectoderm at various positions

**Figure 3:**
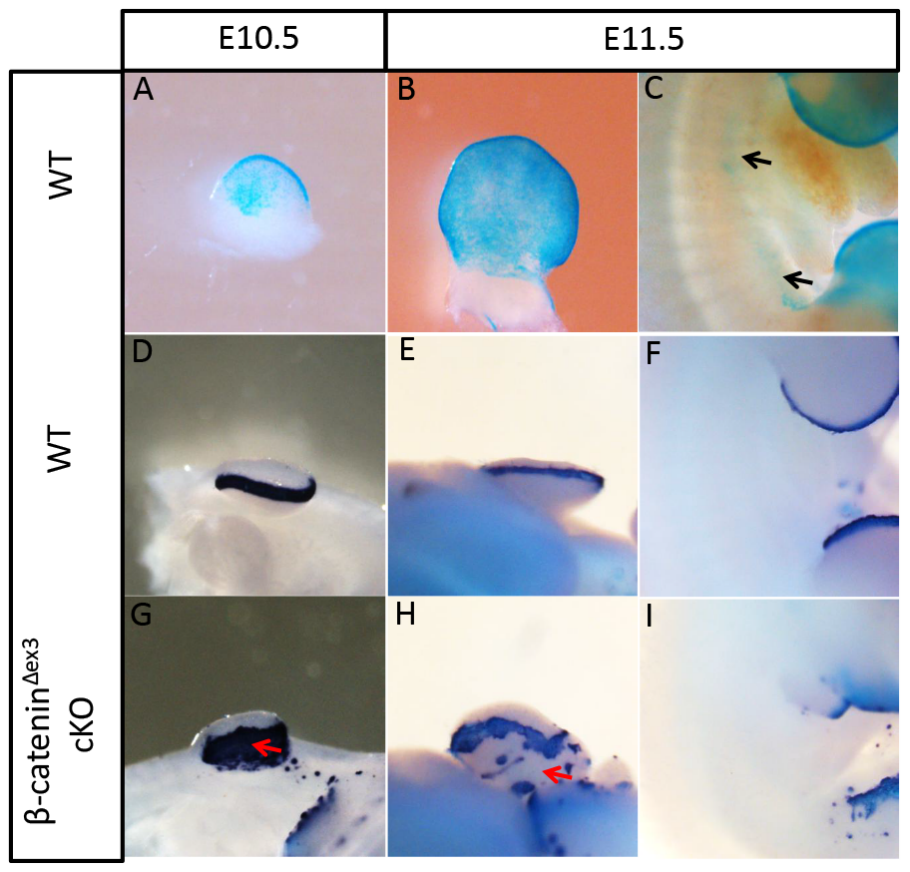
Ectopic expression of Fgf8 diminished with time. (A–C) Activity of Msx2-cre persisted in the ventral forelimb ectoderm and inter-limb flank from E10.5 to E11.5. (D, E, G and H) Compared with E10.5, ectopic expression of Fgf8 in the ventral forelimb ectoderm was significantly reduced at E11.5 (red arrowheads). (F and I) Ectopic expression of Fgf8 disappeared along the inter-limb flank.

In the limb ectoderm, β-catenin activation is sufficient to directly induce the expression of Fgf8 [9], [21]. However, when investigating the expression of Fgf8 in embryos at E11.5, we observed that most of the ectopic Fgf8 expression was diminished or lost, compared with expression in E10.5 embryos (Fig. 2B and E, 3D–I). The observed decrease in expression was not likely caused by attenuated β-catenin activation because Msx2-cre activity was maintained in these regions (Fig. 3A–C).

### Abnormal β-catenin activity affected AVBW closure

**Figure 4:**
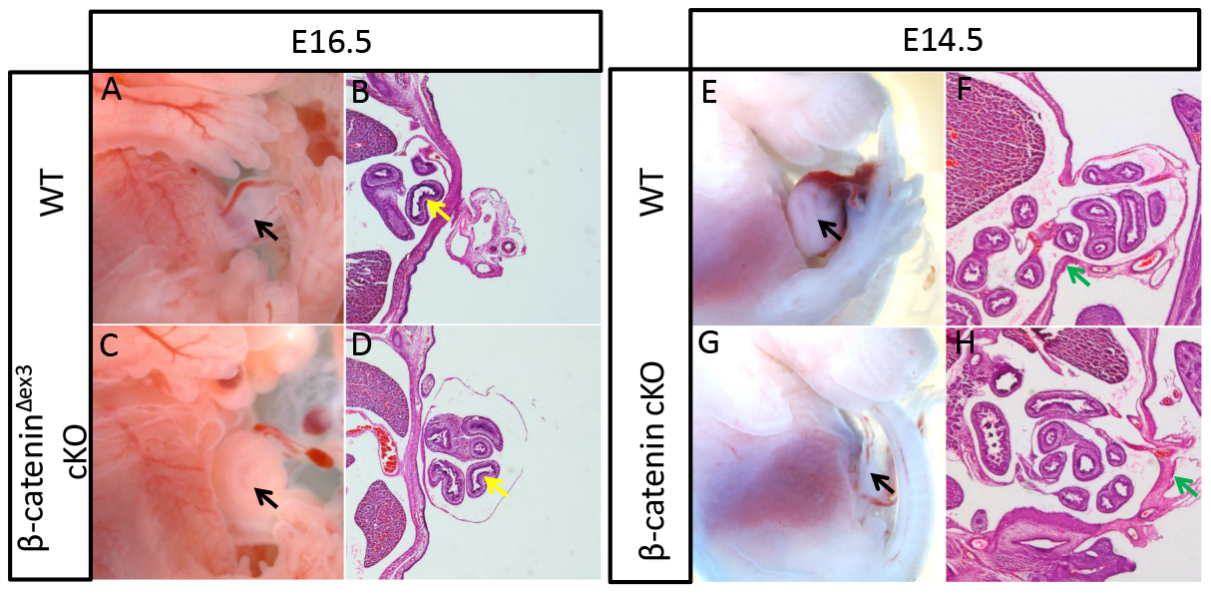
The abnormal level of β-catenin affected AVBW closure. (A and C) a whole-mount view of the abdomen revealed that in severely affected Msx2-cre; β-catenin^Δex3^ mutants, AVBW closure had not been completed at E16.5, at which stage the AVBW of normal littermates has already closed at the abdominal midline (black arrowheads). (B and D) Sagittal view of the abdominal center. Yellow arrowheads indicate the intestines. (E–H) At E14.5, when obvious umbilical ring structures were present in the abdomen of normal embryos, the AVBW was closed in severely affected Msx2-cre; β-catenin mutants (black arrowheads), as revealed by the enclosure of the intestines by the body wall (F and H). Green arrowheads indicate the body wall.

In addition to the phenotypes mentioned above, approximately 30% (10/29) of Msx2-cre; β-catenin^Δex3^ mutant also exhibited defect in AVBW closure. By E16.5, when the secondary body wall had fused in the ventral midline, these mutant embryos still showed obvious umbilical ring structures, in which intestines could be clearly identified (Fig. 4A–D). However, the impairment did not appear to be permanent because no significant AVBW defects were observed in these mutants after birth (n = 15). The mutants that displayed AVBW defects generally had more outgrowths in the abdominal region and most of these mutants had fused forelimb-hindlimb structures (Fig. S3). We hypothesized that when more LPM cells were specified to form limb or ectopic outgrowths, the remaining AVBW-forming LPM that was not sufficient to guarantee a normal rate of AVBW closure. We subsequently investigated whether disrupted limb formation promoted AVBW closure. When we deleted β-catenin using Msx2-cre in the ectoderm, hindlimbs were uniformly absent and forelimb truncation varied in extent. In Msx2-cre; β-catenin cKO mutants with severely truncated forelimbs (7/24), the AVBW was prematurely closed at E14.5, whereas in all control embryos, clear umbilical rings, containing intestines, could be observed in the middle of abdomen (Fig. 4E–H), suggesting a counterbalance between limb formation and AVBW closure.

## Discussion

In this study, we analyzed limb-forming potential at various positions along the cranial-caudal axis of the mouse embryos by activating ectodermal β-catenin, which was sufficient to trigger the expression of Fgf8 in inter-limb LPM and in the head and tail regions. Ectopic activity of β-catenin/Fgf8 resulted in accessory limb formation within the inter-limb region, while in the head and tail, β-catenin/Fgf8 induced outgrowths with Msx1 and Shh expression within the underlying mesenchyme. In the abdominal region, the ectopic formation of limbs was correlated with defective AVBW closure, whereas disrupted limb growth promoted premature AVBW closure, which suggested that limb formation may affect AVBW closure.

During limb initiation in chicks, the Wnt2b/8c/β-catenin pathway in the LPM triggers mesodermal expression of Fgf10, inducing the activation of the overlying ectoderm to synthesize Wnt3a. The ectodermal Wnt3a/β-catenin pathway initiates the expression of Fgf8, which is required for limb bud induction [8]. In mice, whether such a Wnt-Fgf-Wnt signal relay crossing the two germ layers is preserved during limb initiation remains unclear, but our results suggested ectodermal Wnt/β-catenin was sufficient to induce limb formation in the flank region.

In chick embryos, ectopic Fgf8 can be induced by exogenous Fgf7 along the rostral-caudal axis of the embryo back, but this induction only occurred posterior to the 10^th^ somite [10]. Our analysis in mice showed that more anterior positions, such as the head region, also possessed the ability to express ectopic Fgf8 in response to Wnt3a/β-catenin stimulation. The mesenchyme in these positions could bulge outwards by Fgf8 induction, mimicking the event of normal limb bud initiation. However, although these extra outgrowths displayed some molecular similarity with limb buds, only the outgrowths immediately adjacent to normal limbs developed molecular markers for distal limb patterning. These results indicated that only the presumptive limb-forming region can provide sufficient guidance for development of a true limb a real limb whereas other positions may just partially reserve limb-forming ability.

In addition, our analysis revealed that sustained β-catenin activation was insufficient to maintain the ectopic expression of Fgf8, indicating thatadditional factors are necessary to support continuous Fgf8 expression in the ectoderm. During the development of normal limb, either the persistence of the AER or ectodermal Fgf expression for an appropriate duration is required to maintain limb growth, i.e., longer Fgf exposure produces more tissue [6]. Thus, the relatively small size of outgrowths in Msx2-cre; β-catenin^Δex3^ mutants is highly possibly because of the short duration of Fgf signal stimulus as the ectopic expression of Fgf8 rapidly diminished.

Limb development in both chick and mouse embryos occurs considerable earlier than AVBW closure, which suggests that in the LPM, limb formation may be prioritized over AVBW formation. In addition, on the basis of our results we speculate that the progenitor pool in the LPM that could be directed to form both types of tissues is limited. When ectopic limbs formed, the pool of progenitors remained to form AVBW was insufficient to support a growth rate that would result in normal closure. By contrast, when limb formation was disrupted, more LPM progenitors were directed to form the AVBW, which resulted in premature closure. This speculation also explains that the limb phenotype of the Msx2-cre; β-catenin^Δex3^ mutants, which was characterized by a hyperplastic autopod and a hypomorphic zeugopod, i.e., when limited limb progenitors were guided to form extra autopod elements, the progenitors remaining for zeugopod formation correspondingly decreased. A similar model has been proposed to explain limb pattern establishment [22], which further supports our speculative explanation of the counterbalance between limb formation and AVBW closure in mice.

## Supporting Information

Figure S1 **Msx2-cre activity was presented at different positions along the mouse embryos at E10.5.** (A) Cre activity in the head ectoderm. (B) Cre activity along the inter-limb flank. (C) Cre activity in the tail region. FL, forelimb; HL, hindlimb.
(TIF)

Figure S2 **Skeletal analysis of the skull and limbs in controls and Msx2-cre; β-catenin^Δex3^ mutants at E16.5.** (A and D) Lateral view of the normal and mutant skulls. The parietal bone was nearly absent in mutants (black arrowhead). (B, C, E and F) Compared with control embryos, mutants had shortened zeugopods with disrupted mineralization (black arrowheads), and the autopods were hyperplastic with severe polydactyly. FL, forelimb; HL, hindlimb. (G–I) Transverse sections of distal autopods. Mutants showed pre-axial polydactyly and post-axial polydactyly (black arrowheads), as well as dorsal and ventral polydactyly (red arrowheads).
(TIF)

Figure S3 **Lateral view of controls and Msx2-cre; β-catenin^Δex3^ mutants at E16.5.** (A and B)The forelimb and hindlimb of severely affected mutants were fused by extra tissue in the distal region. As a result, inter-limb AVBW formation was considerably impaired. (C) The relative length of the flank region between forelimb and hindlimb, measured in three pairs of embryos was measured. Student's *t-*test was used to calculate statistical significance; error bars show standard deviation. AVBW, abdominal ventral body wall; FL, forelimb; HL, hindlimb; *, *P*<0.05.
(TIF)
