# Supplementary figures and images for: Constitutive Activation of Ectodermal β-Catenin Induces Ectopic Outgrowths at Various Positions in Mouse Embryo and Affects Abdominal Ventral Body Wall Closure
Source: PLoS One. 2014 Mar 19;9(3):e92092. doi: 10.1371/journal.pone.0092092 (PMC3960177; doi:10.1371/journal.pone.0092092)

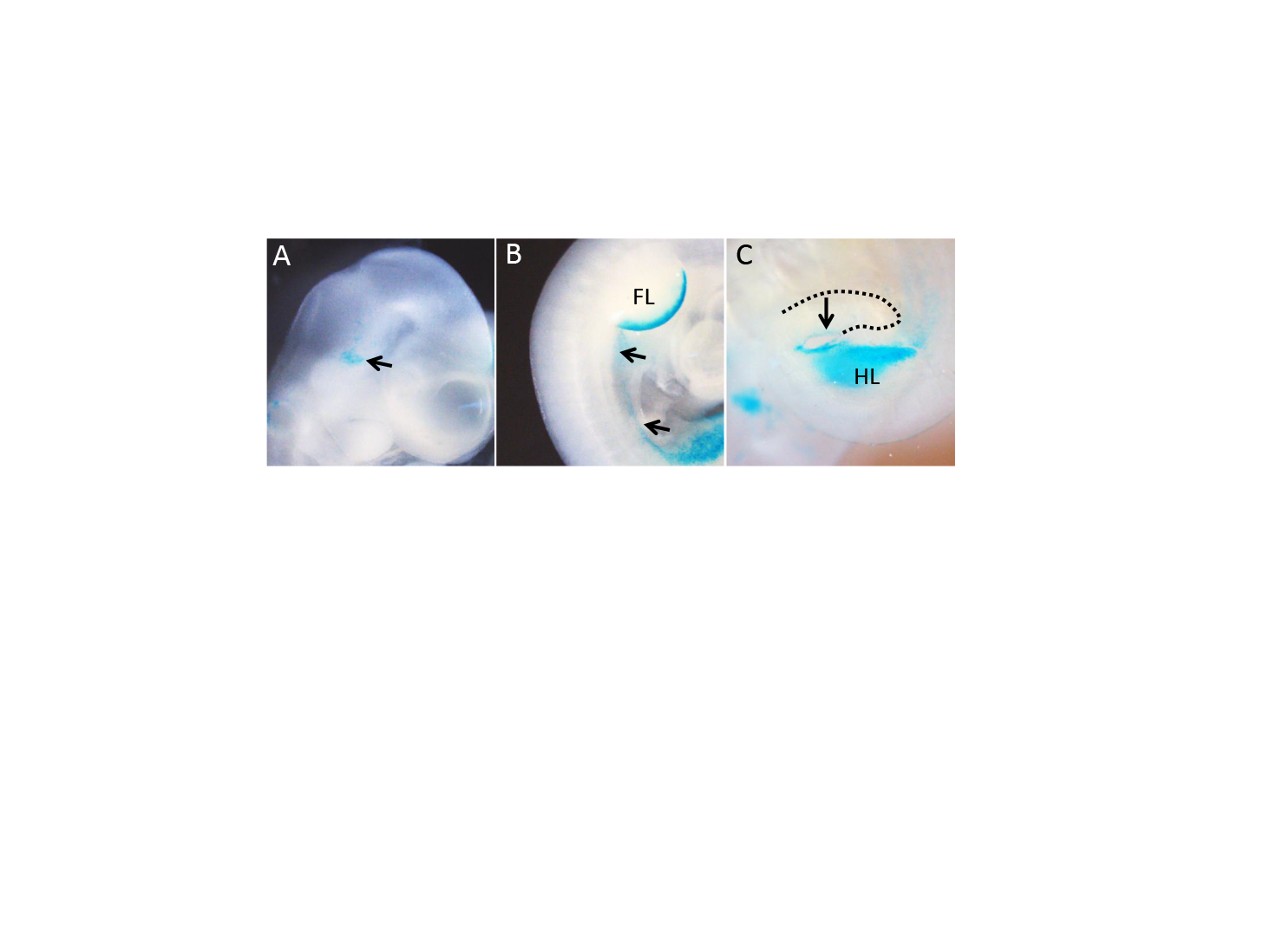

Supplement: Figure S1 — Msx2-cre activity was presented at different positions along the mouse embryos at E10.5. (A) Cre activity in the head ectoderm. (B) Cre activity along the inter-limb flank. (C) Cre activity in the tail region. FL, forelimb; HL, hindlimb. (TIF) [file pone.0092092.s001.tif]

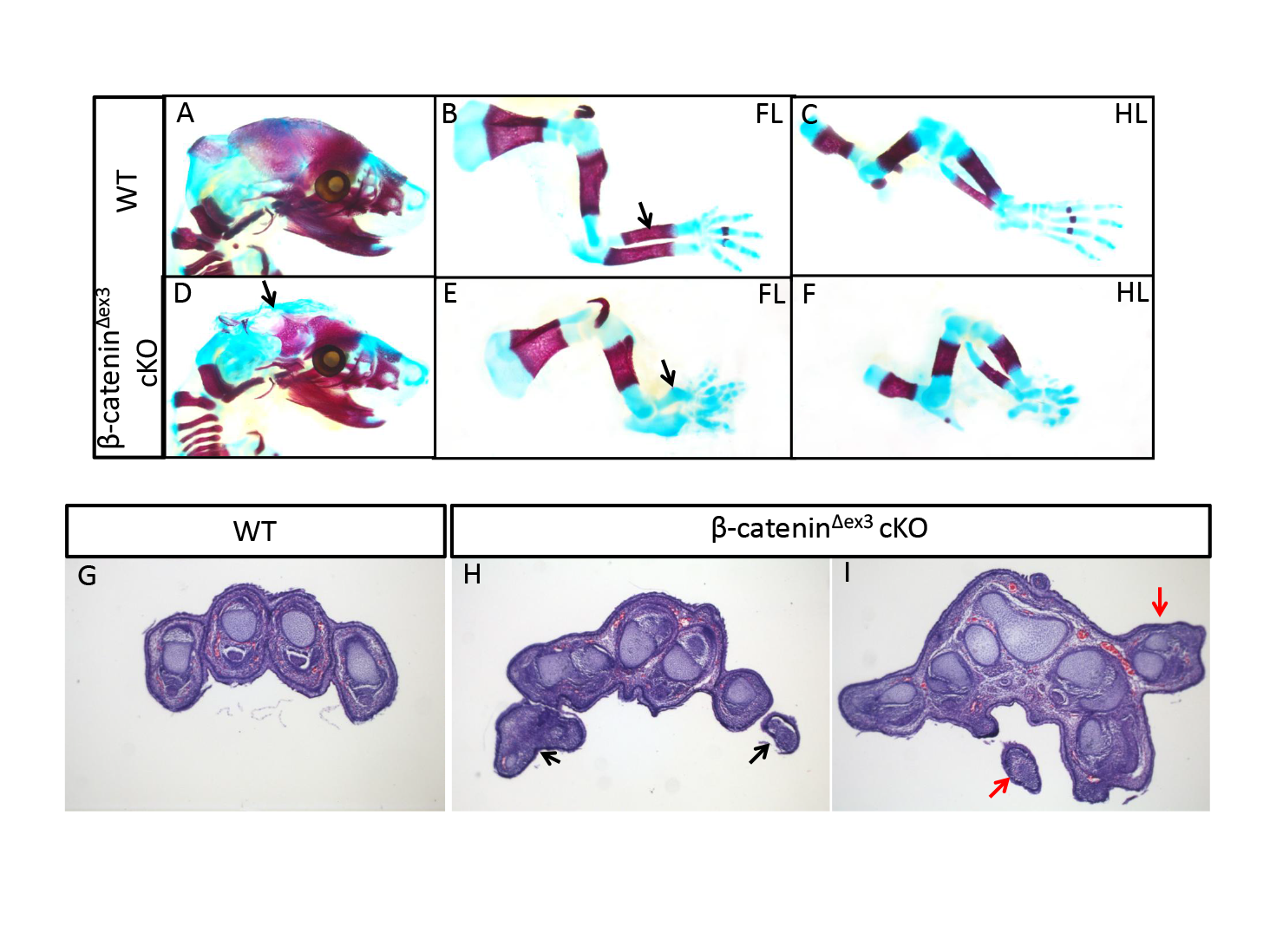

Supplement: Figure S2 — Skeletal analysis of the skull and limbs in controls and Msx2-cre; β-cateninΔex3 mutants at E16.5. (A and D) Lateral view of the normal and mutant skulls. The parietal bone was nearly absent in mutants (black arrowhead). (B, C, E and F) Compared with control embryos, mutants had shortened zeugopods with disrupted mineralization (black arrowheads), and the autopods were hyperplastic with severe polydactyly. FL, forelimb; HL, hindlimb. (G–I) Transverse sections of distal autopods. Mutants showed pre-axial polydactyly and post-axial polydactyly (black arrowheads), as well as dorsal and ventral polydactyly (red arrowheads). (TIF) [file pone.0092092.s002.tif]

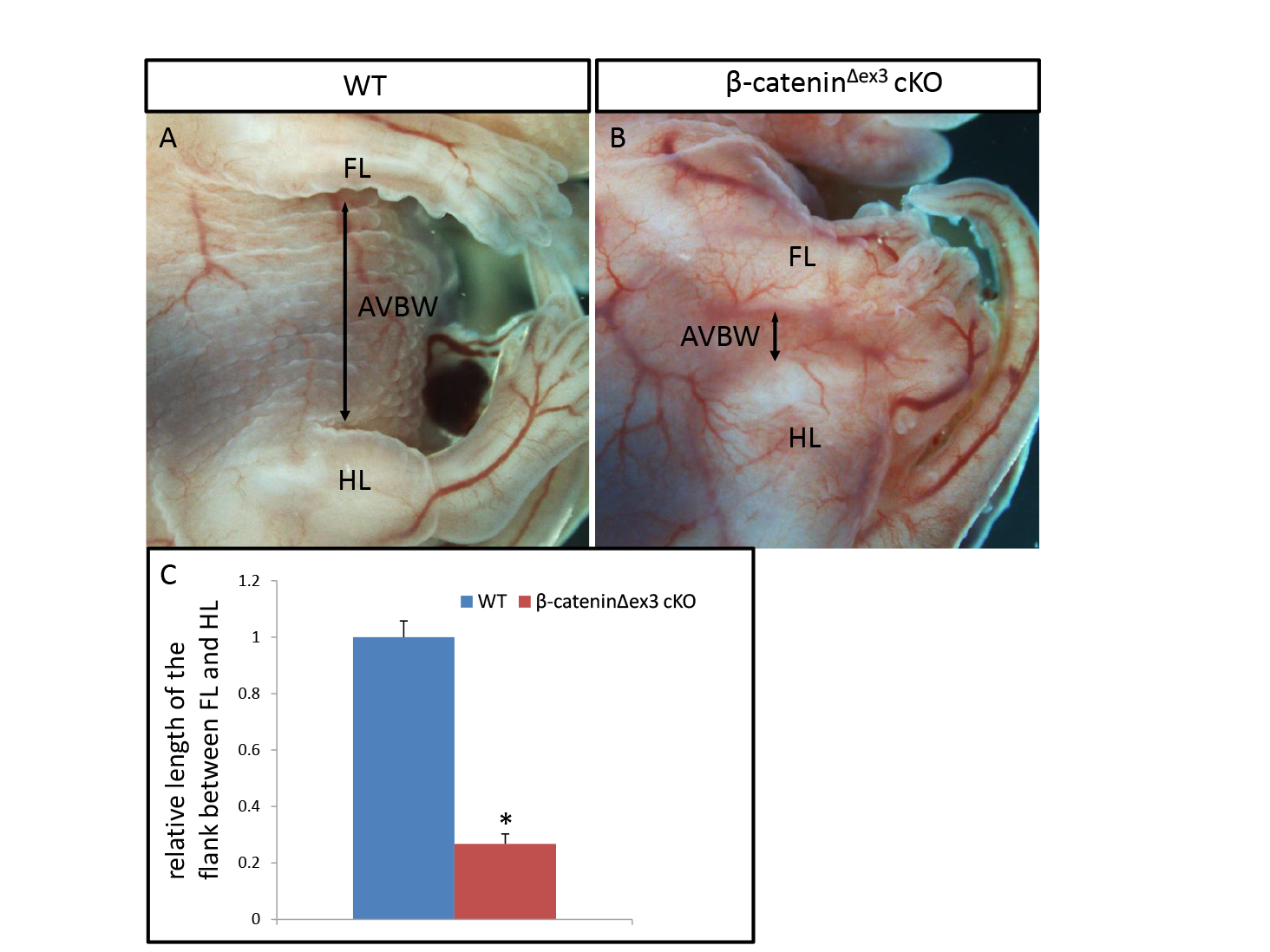

Supplement: Figure S3 — Lateral view of controls and Msx2-cre; β-cateninΔex3 mutants at E16.5. (A and B)The forelimb and hindlimb of severely affected mutants were fused by extra tissue in the distal region. As a result, inter-limb AVBW formation was considerably impaired. (C) The relative length of the flank region between forelimb and hindlimb, measured in three pairs of embryos was measured. Student's t-test was used to calculate statistical significance; error bars show standard deviation. AVBW, abdominal ventral body wall; FL, forelimb; HL, hindlimb; *, P<0.05. (TIF) [file pone.0092092.s003.tif]
